# Long-term drug administration in the adult zebrafish using oral gavage for cancer preclinical studies

**DOI:** 10.1242/dmm.024166

**Published:** 2016-07-01

**Authors:** Michelle Dang, Rachel E. Henderson, Levi A. Garraway, Leonard I. Zon

**Affiliations:** 1 Stem Cell Program and Division of Pediatric Hematology/Oncology, Boston Children's Hospital, Boston, MA 02115, USA; 2 Howard Hughes Medical Institute, Boston, MA 02115, USA; 3 Harvard Medical School, Boston, MA 02138, USA; 4 Department of Medical Oncology, Dana-Farber Cancer Institute, Boston, MA 02215, USA; 5 The Broad Institute of MIT and Harvard, Cambridge, MA 02142, USA

**Keywords:** Adult zebrafish, Oral gavage, Melanoma, Vemurafenib

## Abstract

Zebrafish are a major model for chemical genetics, and most studies use embryos when investigating small molecules that cause interesting phenotypes or that can rescue disease models. Limited studies have dosed adults with small molecules by means of water-borne exposure or injection techniques. Challenges in the form of drug delivery-related trauma and anesthesia-related toxicity have excluded the adult zebrafish from long-term drug efficacy studies. Here, we introduce a novel anesthetic combination of MS-222 and isoflurane to an oral gavage technique for a non-toxic, non-invasive and long-term drug administration platform. As a proof of principle, we established drug efficacy of the FDA-approved BRAF^V600E^ inhibitor, Vemurafenib, in adult zebrafish harboring BRAF^V600E^ melanoma tumors. In the model, adult casper zebrafish intraperitoneally transplanted with a zebrafish melanoma cell line (ZMEL1) and exposed to daily sub-lethal dosing at 100 mg/kg of Vemurafenib for 2 weeks via oral gavage resulted in an average 65% decrease in tumor burden and a 15% mortality rate. In contrast, Vemurafenib-resistant ZMEL1 cell lines, generated in culture from low-dose drug exposure for 4 months, did not respond to the oral gavage treatment regimen. Similarly, this drug treatment regimen can be applied for treatment of primary melanoma tumors in the zebrafish. Taken together, we developed an effective long-term drug treatment system that will allow the adult zebrafish to be used to identify more effective anti-melanoma combination therapies and opens up possibilities for treating adult models of other diseases.

## Abstract (precis)

**Summary:** We have developed the first long-term drug delivery system in the adult zebrafish using oral gavage, and offer a foundation for future preclinical studies of cancer therapeutics in adult zebrafish.

## INTRODUCTION

The zebrafish is an invaluable *in vivo* model for translational oncology because of its adaptability in transgenesis, genome-editing, transplantation and imaging (Suster et al., 2009; Hwang et al., 2013; White et al., 2008). Transgenic zebrafish cancer models can genetically and histopathologically mimic human cancers, making the zebrafish an excellent model for an inexpensive and highly scalable platform for *in vivo* drug testing in a preclinical trial (Ceol et al., 2008; White et al., 2013) Whereas the zebrafish embryo has been used to identify and test novel anti-cancer therapeutics, the technical challenges of drug delivery in adult zebrafish have limited progress in this field.

Whereas chemicals are directly added to the water for treatments in zebrafish embryos and larvae, drug administration in the adult zebrafish is more challenging (Burns et al., 2005; Berghmans et al., 2008). Passive drug delivery methods, including dissolution of the chemical into the water, are severely ineffective and expensive for water-insoluble compounds. Other documented passive techniques include incorporation of the drug into fish feed through coated capsules (Sciarra et al., 2014). In these passive methods, it is difficult to control the concentration of the drug that the zebrafish take up though the gills (Magno et al., 2015). Alternative approaches such as retro-orbital or intraperitoneal injections provide a means to directly administer a controlled concentration of water-insoluble drugs (Pugach et al., 2009; Kinkel et al., 2010). Although these techniques are effective as single administration techniques, long-term and repeated injections often lead to injury and infection.

Oral gavage offers a controlled delivery method without the trauma introduced by invasive injections, potentially allowing for long-term daily treatments. Microgavage in zebrafish larvae using zebrafish microinjection manipulators and stereomicroscopy has been largely successful (Cocchiaro and Rawls, 2013; Goldsmith et al., 2013) However, early attempts at oral gavage in adult zebrafish simply used catheter sheaths attached to pipettes or blunt-tipped gavage syringes (Tysnes et al., 2012; Marie et al., 2012). Advancements in the catheter tubing of gavage apparatus has significantly reduced trauma and injury for single administrations (Collymore et al., 2013). Although the methodology of oral gavage has been previously demonstrated as an effective single-intervention technique, it has yet to be developed into a multi-day, long-term drug efficacy study. Overcoming challenges in drug dosing optimization and anesthesia-related toxicity will be crucial in developing the zebrafish as a cost-efficient means for preclinical drug toxicology and efficacy studies.

The transgenic zebrafish melanoma model expresses human oncogenic mutant BRAF^V600E^ driven by the melanocyte-specific *mitfa* promoter in a *p53^−/−^*-deficient background. These adult zebrafish have normal pigmentation and stripe patterning, and develop primary tumors after many months to a year (Patton et al., 2005). Nacre mutant zebrafish lack pigmentation and stripe patterning resulting from the loss of *mitfa*, the master regulator of the melanocyte lineage, and these zebrafish can never develop melanoma. Similarly, *Tg(mitfa:BRAF^V600E^); p53^−/−^; mitfa^−/−^* zebrafish are primed to develop melanomas once *mitfa* is rescued. The MiniCoopR expression vector is a Tol2-based vector that expresses the *mitfa* minigene driven by the *mitfa* promoter, and drives the expression of a candidate gene of choice also driven by the *mitfa* promoter in cis. Microinjection of the MiniCoopR expression vector into one-cell-staged *Tg(mitfa:BRAF^V600E^); p53^−/−^; mitfa^−/−^* embryos can overexpress a candidate gene of choice in mosaically rescued melanocytes. In this transgenic melanoma model, adult zebrafish develop primary tumors overexpressing control eGFP with a median onset of 18 weeks (Ceol et al., 2011).

The transplantation model utilizes a transparent adult zebrafish as an *in vivo* tool to analyze tumor cell engraftment, proliferation and metastasis. Adult caspers lack pigmentation as a result of mutations in genes *nacre* and *roy*, making this transparent zebrafish a valuable transplantation recipient as it allows for the tracking of engrafted pigmented melanoma tumors (Li et al., 2011; White et al., 2008). Irradiated adult casper zebrafish can either be transplanted intraperitoneally or subcutaneously with zebrafish melanoma cell lines or primary zebrafish melanoma tumors, and develop large pigmented tumors by 10 days post-transplantation. The dark pigmentation of the donor material starkly contrasts with the transparent casper, and allows for direct visualization of not only engraftment and proliferation, but also of tumor size in response to drug treatments.

Here, we provide the first proof-of-principle study promoting the use of oral gavage in long-term drug efficacy studies in adult zebrafish. Developing advancements in the anesthetic protocol allowed for multi-day drug studies and most importantly, we demonstrate oral dosing in the zebrafish model as an effective means to administer a FDA-approved small molecule inhibitor. We treated adult zebrafish harboring BRAF^V600E^-mutant melanoma with the BRAF^V600E^-specific inhibitor, Vemurafenib, and observed an average 65% tumor reduction in the drug-treated cohorts compared with an average 18% tumor growth in the control-treated cohorts. In contrast, application of the drug regimen on adult zebrafish harboring Vemurafenib-resistant melanoma resulted in an average 15% tumor growth in both Vemurafenib and control-treated cohorts. Most importantly, extension of the oral gavage technique as an effective and non-invasive long-term drug delivery strategy provides a platform for the usage of adult zebrafish in preclinical cancer trials.

## RESULTS

### Technical advancements in oral gavage

**Fig. 1.**
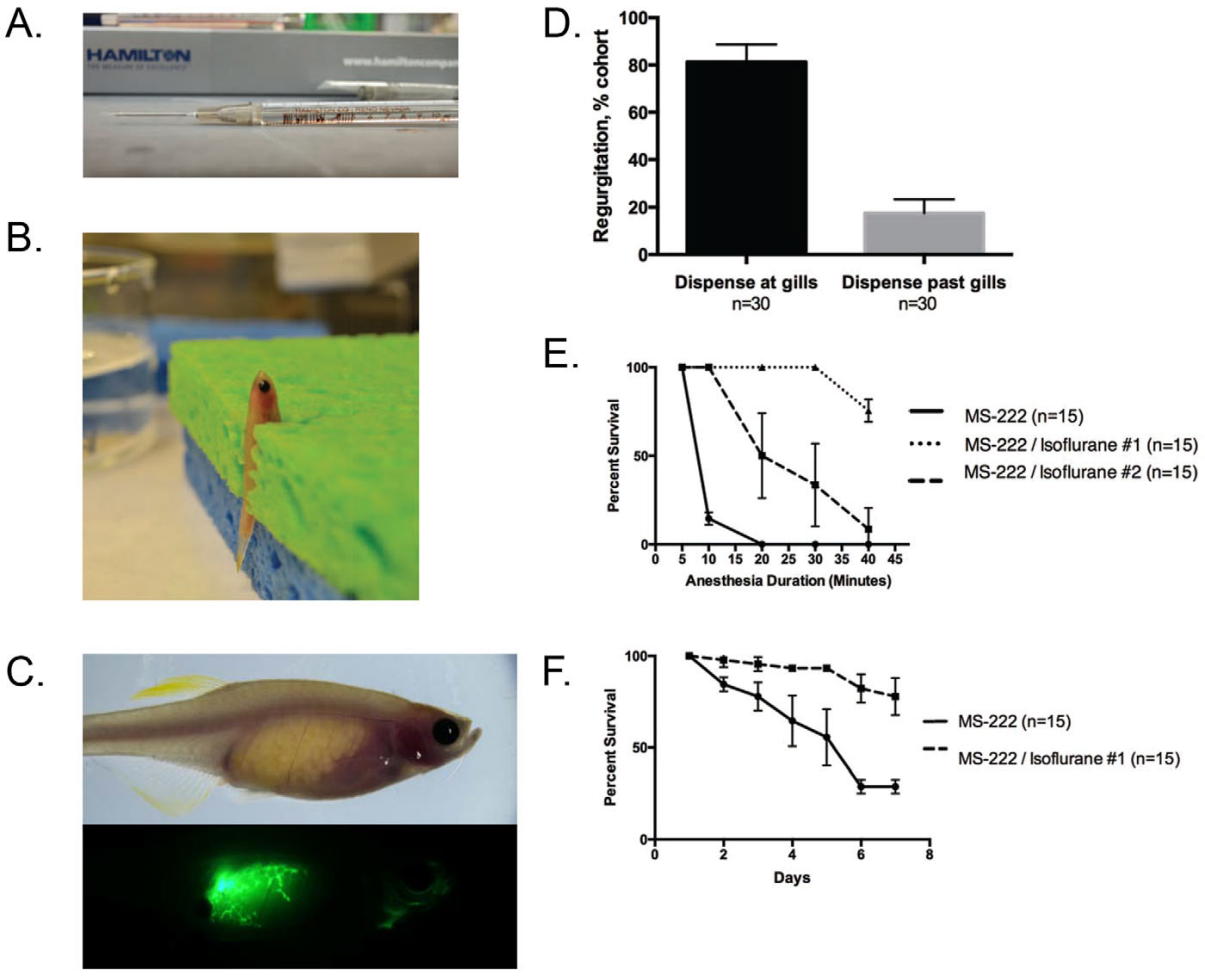
**Technical optimization of oral gavage technique.** (A) The gavage apparatus was constructed from a 10 µl luer-tip Hamilton syringe with a 22 G needle and 22 G soft-tip catheter tubing. (B) A representative zebrafish was anesthetized using a MS-222/isoflurane combination anesthetic and immobilized vertically in a damp sponge with a slit. (C) A representative zebrafish was gavaged with 3 μl fluorescein isothiocyanate-dextran (FITC-dextran) to visualize potential regurgitation via fluorescence microscopy. (D) Zebrafish were gavaged with Phenol Red solution to visualize potential regurgitation. Data represented as mean±s.d. of two replicates. (E) Survival curve for extended exposure to anesthetic solutions. Data represented as mean±s.d. of three replicates. (F) Survival curve for long-term daily exposure to anesthetic solution. Data represented as mean±s.d. of three replicates.

The use of a 10 μl Hamilton syringe allowed for finer control when administering small volumes of solution to the adult zebrafish (Fig. 1A). The zebrafish is vertically immobilized in a damp sponge with the gills exposed (Fig. 1B). In order to rule out the possibility of losing drug volume through regurgitation or passive leakiness through the gills, preliminary studies utilized fluorescent dextran or Phenol Red as a visual indicator of the gavaged solution. Adult zebrafish were gavaged with 3 μl of FITC-dextran and immediately observed for a 20-min period by fluorescence microscopy. In this time frame, the fluorescent solution did not expel through either the gills or the mouth of the zebrafish and remained largely in the intestinal lumen (Fig. 1C). To address the possibility of the drug leaking via the gills or being regurgitated during the recovery process, adult caspers were gavaged with a Phenol Red solution. The first cohort of fish was deliberately gavaged at the level of gills and resulted in the Phenol Red solution diffusing out of the zebrafish. The second group of fish was gavaged past the level of the gills and resulted in no leakage or regurgitation during the recovery time. Taken together, these results demonstrate that the gavaged liquid remains in the intestinal lumen and is neither regurgitated nor diffused out of the gills (Fig. 1D).

Although oral gavage has been previously demonstrated as an effective single-intervention technique, challenges in MS-222-related toxicity prevented the gavage application being used in long-term drug studies. Standard anesthetic doses of MS-222 resulted in significant death when adult zebrafish are exposed for greater than five minutes; furthermore, repeated daily three-minute exposure of MS-222 alone resulted in steadily decreasing survival of the cohort (Fig. 1E,F).

To address the challenge of MS-222 toxicity, addition of a second anesthetic, isoflurane, significantly improved survival. The MS-222/isoflurane combination anesthetic significantly improved survival of adult zebrafish anesthetized for up to 40 min compared with the MS-222-only anesthetic. Most importantly, the MS-222/isoflurane combination anesthetic is significantly more tolerable in repeated daily three-minute exposures (Fig. 1E,F). The application of this combination anesthetic minimized trauma and injury and allowed for long-term interventions in the adult zebrafish.

### Zebrafish melanoma cell line (ZMEL1) engraft in casper recipients

**Fig. 2.**
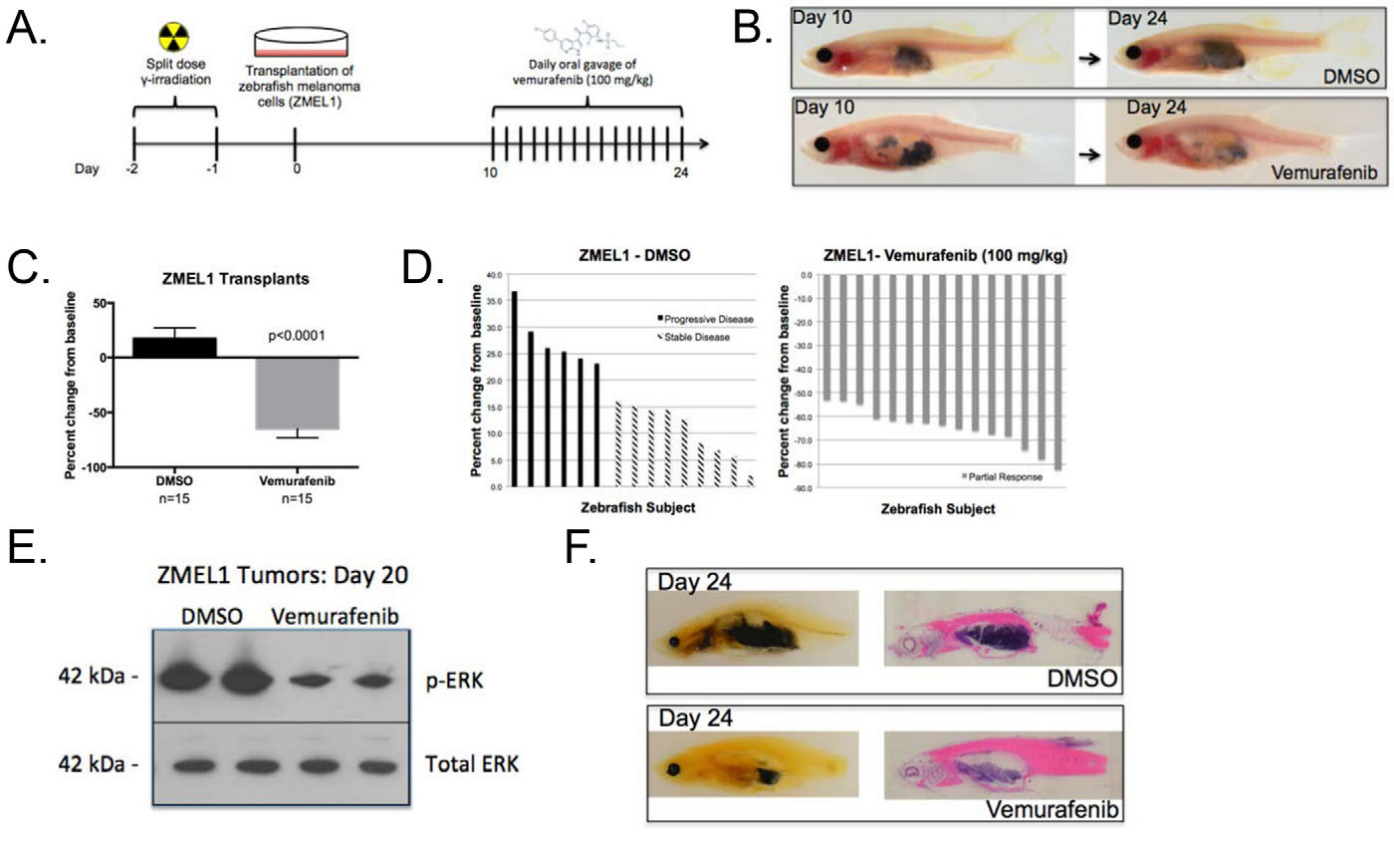
**BRAF^V600E^ inhibitor treatment of zebrafish melanoma cell line (ZMEL1) transplants.** (A) Experimental workflow: MHC caspers were exposed to 30 Gy split-dose of sub-lethal irradiation day –2 and –1 prior to transplantation on day 0. MHC caspers were transplanted with 500,000 zebrafish melanoma cells (ZMEL1) and allowed a 10-day period for melanoma engraftment and proliferation. A two-week regimen of daily oral gavage of DMSO-control or 100 mg/kg Vemurafenib began on day 10. (B) The top panel shows a representative ZMEL1-transplanted zebrafish treated with DMSO control over a two-week treatment regimen. The bottom panel shows a representative ZMEL1-transplanted zebrafish treated with 100 mg/kg Vemurafenib over a two-week treatment regimen. (C) Average percent change from baseline of tumor area based on pigmentation in a cohort with *n*=15 in each treatment arm. Two-tailed paired *t*-test was performed for statistical analysis. Data represented as mean±s.d. of three replicates. (D) Waterfall plot depicting the range of response for the experimental cohorts, DMSO and Vemurafenib. The response was quantified by percent change of tumor area from baseline. (E) ZMEL1 tumors from DMSO control (lanes 1 and 2) and Vemurafenib-treated (lanes 3 and 4) cohorts were isolated at day 20. MAPK activity was measured via phosphorylated-ERK with total ERK as a loading control. (F) Paraffin section (left) and H&E stain (right) of representative zebrafish within a DMSO (top panel) or Vemurafenib (bottom panel) cohort at day 24.

In our studies, we assessed drug efficacy of Vemurafenib in various adult zebrafish melanoma models harboring BRAF^V600E^-dependent tumors as a proof-of-principle for the oral gavage technique as a long-term drug administration method. The transplantation model allows for large, homogeneous cohorts of tumor-bearing adult zebrafish to be generated for drug efficacy studies. Transplant recipients were immunosuppressed through 30 Gy of split-dose sub-lethal γ-irradiation two days prior to the transplantation to prevent rejection of the donor material (Fig. 2A). ZMEL1 melanoma cells were harvested, counted and resuspended in PBS with a final volume of 5 μl per zebrafish. To establish engraftment of zebrafish melanoma cell lines in the irradiated casper recipients, increasing cell dosages were transplanted into the intraperitoneal cavity with a 10 μl Hamilton syringe. Visible tumor engraftment, as measured by pigmentation, was visible as early as 7 days post-transplantation and large tumors spanning the intraperitoneal cavity developed by 10 days post-transplantation. Whereas casper recipients transplanted with 10,000 ZMEL1 melanoma cells only developed small, sparse tumors in the intraperitoneal cavity, casper recipients transplanted with 500,000 ZMEL1 melanoma cells developed a large tumor spanning the entire intraperitoneal cavity at 10 days post-transplantation (Fig. S1). Alternative zebrafish melanoma cell lines, such as eGFP-121.1 and eGFP-121.2 (data not shown) yielded consistent results. This demonstrates the engraftability of cultured zebrafish melanoma cell lines into irradiated zebrafish recipients.

### Assessment of Vemurafenib drug efficacy via oral gavage in transplanted ZMEL1 melanoma tumors

Following transplantation and tumor engraftment, the transplanted zebrafish recipients were divided into two cohorts of 30 subjects per treatment arm at day 10. Each recipient was anesthetized with a MS-222/isoflurane-buffered solution until the zebrafish was fully immobilized. Following anesthesia, the adult zebrafish was transferred and immobilized vertically into a damp sponge for oral gavage. Each zebrafish was positioned with its ventral side into the crevice of the sponge holster and its dorsal side facing the researcher. The zebrafish was positioned such that its mouth was protruding from the edge of the sponge while its gills were gently situated in the crevice of the sponge (Fig. 1B). The Hamilton syringe was held vertically at a 5-10° angle towards the researcher and gently inserted into the oral cavity of the zebrafish. The syringe was retracted and repositioned if the researcher felt any contact or resistance. Once the catheter tip passed the gills, a 3 μl volume of either Vemurafenib (100 mg/kg) dissolved in DMSO or a DMSO-only control was dispensed into the cavity of the zebrafish, and the Hamilton syringe was gently retracted. Immediately after gavage, the zebrafish was returned to a sterile isolation tank with fresh fish water and monitored for recovery, generally occurring within 1-2 min. This demonstrates the minimally invasive nature of the oral gavage technique.

This oral gavage process was repeated once a day for two weeks (Fig. 2A). As demonstrated by a representative zebrafish from each experimental arm (Fig. 2B), daily oral gavage of Vemurafenib (100 mg/kg) significantly reduced tumor burden (*P*<0.001). We observed an average 65% tumor reduction (Fig. 2C) in the drug-treated zebrafish compared with the control-treated zebrafish. The area of the pigmented tumor was quantified with digital calipers and measured pre-drug treatment at 10 days post-transplantation, and post-drug treatment at 24 days post-transplantation. In all zebrafish subjects treated with Vemurafenib, we observed a residual pigmented mass located at the site of injection. This pigmented mass persisted throughout the entire 2-week drug regimen. Consequently, we observed a maximal 82% tumor reduction and according to the criteria of the Response Evaluation Criteria in Solid Tumors (RECIST) algorithm, version 1.1, zebrafish subjects exhibited a significant, but partial, response to Vemurafenib (Nishino et al., 2010). Approximately 13.3% (4 out of 30 subjects) mortality was observed in both the control and drug-treated groups, and occurred within the first two days of treatment (Fig. 2D). Furthermore, as an additional evaluation of drug efficacy we excised the tumor from DMSO control and drug-treated cohorts at day 20 and isolated protein to assess MAPK activity via western blot. Vemurafenib-treated samples had a lower amount of phosphorylated-ERK1/2, suggesting inhibition of MAPK activity (Fig. 2E). Finally, histological analysis demonstrated zebrafish treated with DMSO were heavily tumor burdened compared with zebrafish treated with Vemurafenib (Fig. 2F). This is the first demonstration of drug efficacy of Vemurafenib against BRAF^V600E^-driven melanoma tumors in the adult zebrafish. More importantly, this establishes oral gavage as an effective means to repeatedly deliver chemicals to the adult zebrafish.

**Fig. 3.**
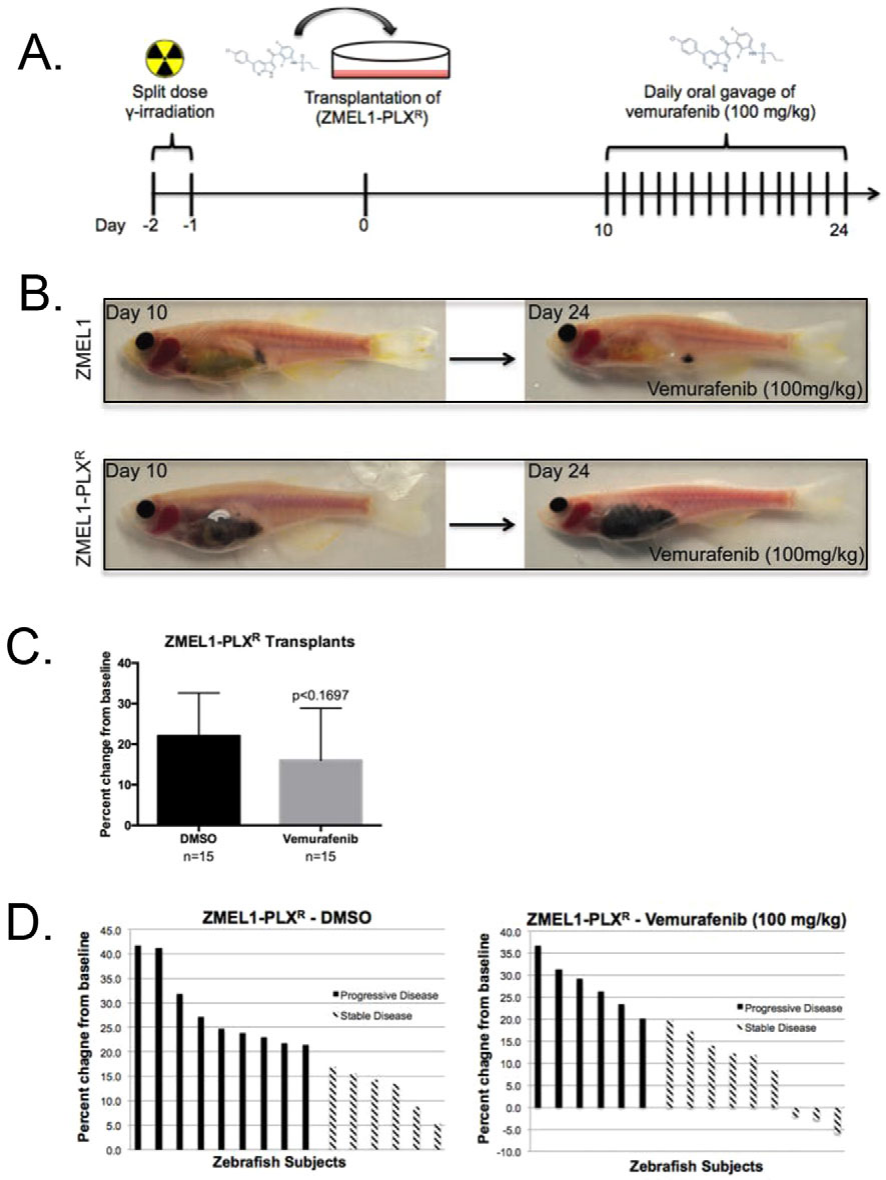
**BRAF^V600E^ inhibitor treatment of drug-resistant zebrafish melanoma cell line (ZMEL1-PLX^R^) transplants.** (A) Experimental workflow: MHC caspers were exposed to 30 Gy split-dose of sub-lethal irradiation day –2 and –1 prior to transplantation on day 0. ZMEL1 cells were exposed to low-dose (50 μM) Vemurafenib in culture for 4 months to select a drug-resistant population, named ZMEL-PLX^R^. MHC caspers were transplanted with 500,000 ZMEL-PLX^R^ cells at day 0. Following a 10-day engraftment and proliferation period, the transplanted zebrafish began a two-week regimen of daily oral gavage of DMSO control or 100 mg/kg Vemurafenib. (B) The top panels show a representative zebrafish transplanted with drug-naïve ZMEL1 cells in the intraperitoneal cavity at day 10 and the right panel shows the same zebrafish after 2 weeks of daily oral gavage of 100 mg/kg Vemurafenib. The bottom panels show a representative zebrafish transplanted with drug-resistant ZMEL-PLX^R^ cells in the intraperitoneal cavity at day 10 and the right panel shows the same zebrafish after 2 weeks of daily oral gavage of 100 mg/kg Vemurafenib. (C) Average percent change from baseline of tumor area based on pigmentation in a cohort with *n*=15 in each treatment arm. Two-tailed paired *t*-test was performed for statistical analysis. Data represented as mean±s.d. of three replicates. (D) Waterfall plots depict the range of response for the experimental cohorts, DMSO and Vemurafenib. The response was quantified by percent change of tumor area from baseline.

In developing a drug treatment model, we were interested in demonstrating the range of drug response that can be detected using the transplant model. ZMEL1 melanoma cells were exposed to low doses of Vemurafenib (50 nM) over a 4-month period to select for a drug-resistant population. The Vemurafenib-resistant ZMEL1-PLX^R^ melanoma cells were transplanted into immunosuppressed casper recipients and allowed to engraft and proliferate for a period of 10 days. Daily oral gavage of 100 mg/kg of Vemurafenib began at day 10 post-transplantation and the treatment regimen persisted for 2 weeks (Fig. 3A). In contrast to the caspers transplanted with drug-naïve ZMEL1 melanoma cells, caspers transplanted with drug-resistant ZMEL1-PLX^R^ line did not respond to 100 mg/kg Vemurafenib via oral gavage (Fig. 3B). In these ZMEL1-PLX^R^ transplants, there was no significant difference between DMSO- or Vemurafenib-treated tumors (Fig. 3C). At the end of the treatment time course, we observed an average 22% and 16% increase in tumor burden in the DMSO-treated recipients and the Vemurafenib-treated recipients, respectively (Fig. 3D). This demonstrates that pigmented Vemurafenib-resistant tumors grew in the intraperitoneal cavity in the presence of Vemurafenib and that the drug treatment model in adult zebrafish using oral gavage can recapitulate *in vitro* drug responses.

### Assessment of Vemurafenib drug efficacy via oral gavage in transplanted primary melanoma

**Fig. 4.**
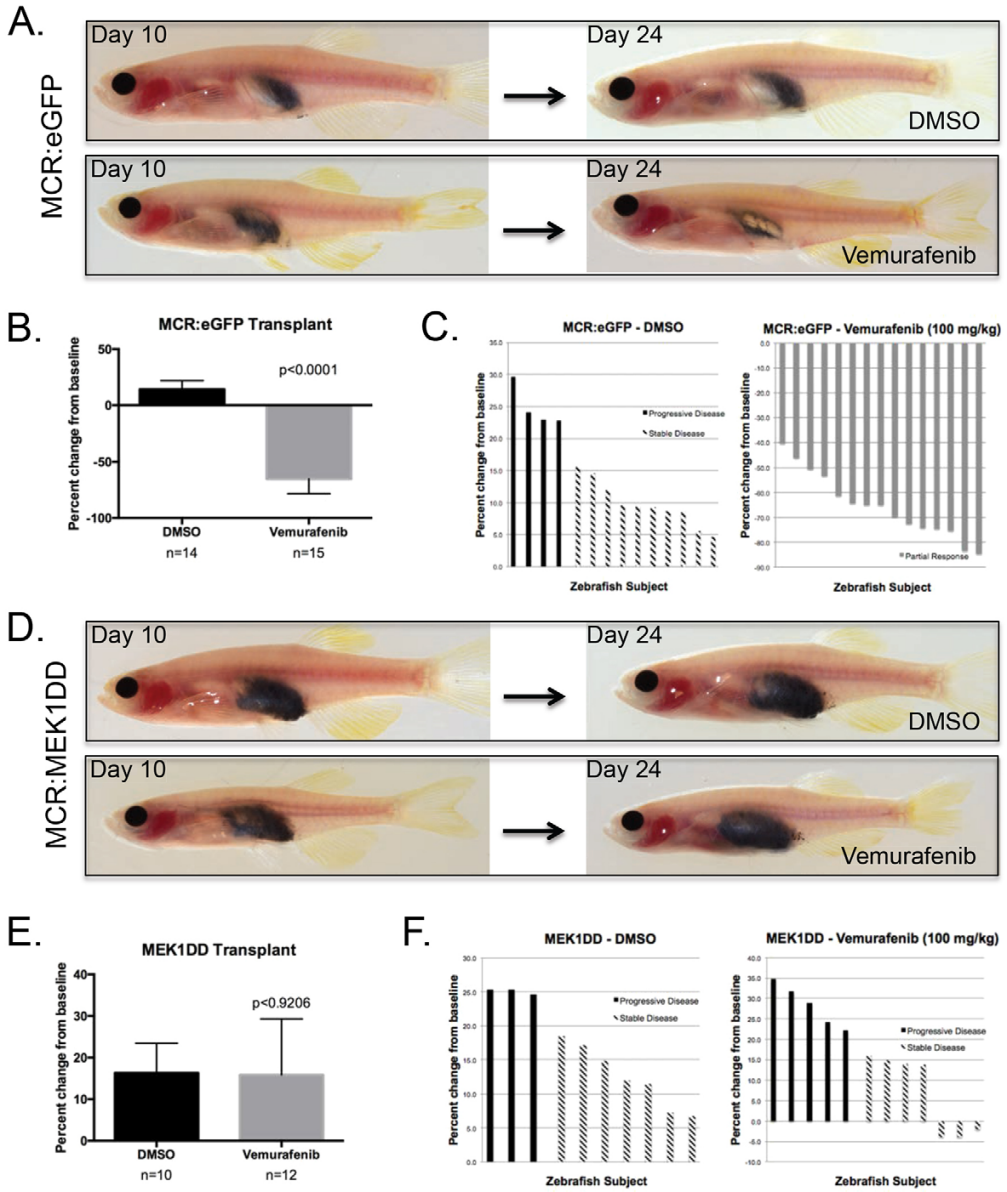
**BRAF^V600E^ inhibitor treatment of primary zebrafish melanoma transplants.** (A) MHC caspers were transplanted with MiniCoopR:eGFP control melanoma tumors. The top panel shows the tumor burden of a representative zebrafish prior to DMSO treatment at day 10 and after a 2-week treatment with DMSO at day 24. The bottom panel shows the tumor burden of a representative zebrafish prior to Vemurafenib treatment at day 10 and after a 2-week treatment regimen with Vemurafenib at day 24. (B) Average percent change from baseline of tumor area based on pigmentation in a cohort with *n*=14 or *n*=15 in the DMSO or Vemurafenib-treated arm, respectively. Two-tailed paired *t*-test was performed for statistical analysis. Data represented as mean±s.d. of three replicates. (C) Waterfall plots depict the range of response for the experimental cohorts, DMSO and Vemurafenib. The response was quantified by percent change of tumor area from baseline. (D) MHC caspers were transplanted with MiniCoopR:MEK1DD melanoma tumors. The top panel shows the tumor burden of a representative zebrafish prior to DMSO treatment at day 10 and after a 2-week treatment with DMSO at day 24. The bottom panel shows the tumor burden of a representative zebrafish prior to Vemurafenib treatment at day 10 and after a 2-week treatment regimen with Vemurafenib at day 24. (E) Average percent change from baseline of tumor area based on pigmentation in a cohort with *n*=10 or *n*=12 in the DMSO or Vemurafenib-treated arm, respectively. Two-tailed paired *t*-test was performed for statistical analysis. Data represented as mean±s.d. of two replicates. (F) Waterfall plots depict the range of response for the experimental cohorts, DMSO and Vemurafenib. The response was quantified by percent change of tumor area from baseline.

The use of oral gavage in the melanoma transplant model can be extended beyond the use of melanoma cell lines to include the use primary melanoma tumors. Primary tumors are heterogeneous and various clones can potentially have differing degrees of drug sensitivity. These primary tumors stem from *Tg(mitfa:BRAF^V600E^); p53^−/−^; mitfa^−/−^*+miniCoopR zebrafish and can better represent *de novo* melanoma. In the transplantation model, Vemurafenib-sensitive primary zebrafish BRAF^V600E^ mutant melanoma tumors derived from transgenic zebrafish overexpressing eGFP though the MiniCoopR Tol2-based expression were homogenized, resuspended in PBS, and transplanted intraperitoneally into irradiated adult casper zebrafish. The transplanted zebrafish were monitored daily for infection, and allowed 10 days for transplanted cells to fully engraft and grow in the intraperitoneal cavity. At 10 days post-transplantation, the fish began a 2-week regimen of daily oral gavage of 100 mg/kg Vemurafenib or DMSO control. Representative zebrafish from each experimental arm demonstrated significant Vemurafenib-dependent decrease in tumor burden (Fig. 4A). Recipients treated with Vemurafenib showed an average 65% decrease in tumor burden, whereas recipients treated with DMSO control showed an average 14% increase in tumor burden (Fig. 4B,C). In contrast, BRAF^V600E^-driven primary melanoma tumors overexpressing constitutively active MEK1DD though the MiniCoopR Tol2-based expression vector are resistant to the Vemurafenib treatment course. Representative recipients from each experimental arm demonstrate tumor growth in the presence of either DMSO or Vemurafenib (Fig. 4D). Both experimental arms showed an average 16% increase in tumor burden at the end of the treatment time course (Fig. 4E,F). This is the first demonstration of Vemurafenib efficacy on zebrafish primary melanoma *in vivo*.

### Assessment of Vemurafenib drug efficacy via oral gavage in primary transgenic zebrafish melanoma

**Fig. 5.**
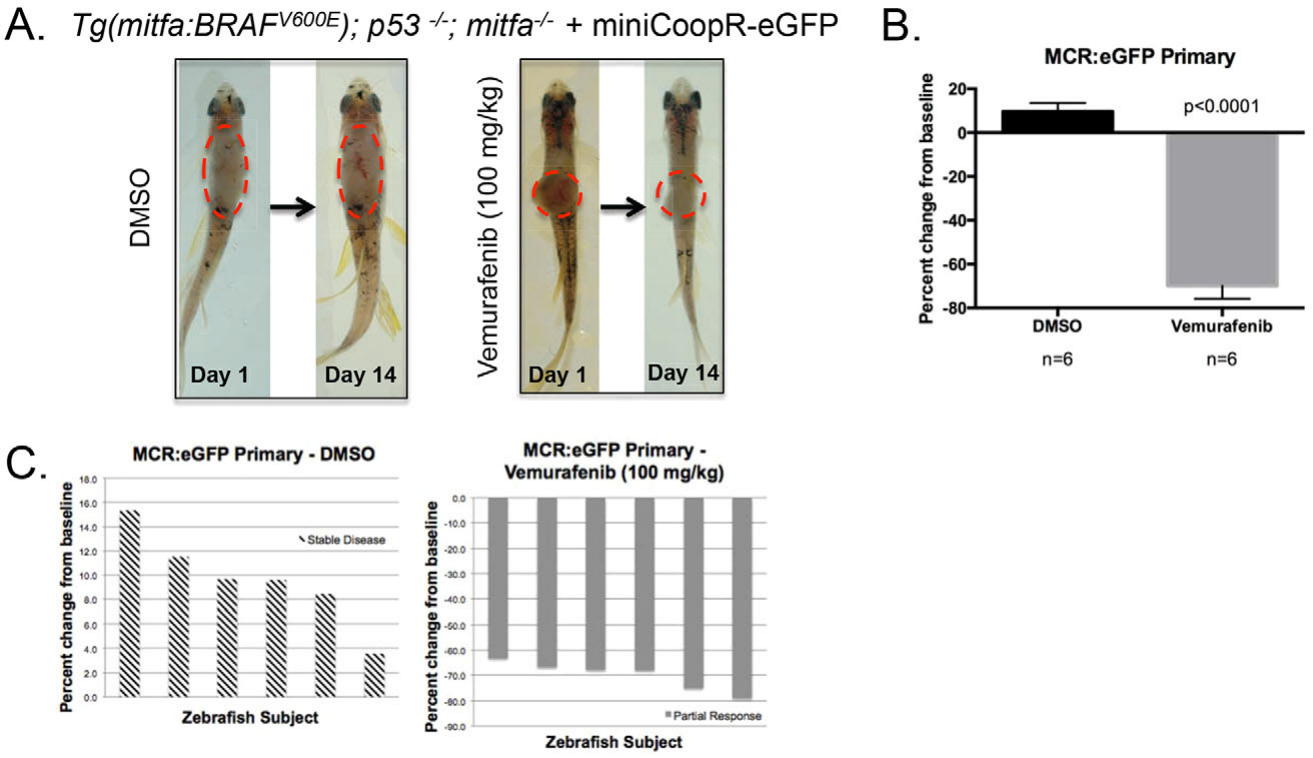
**BRAF^V600E^ inhibitor treatment of primary zebrafish melanoma tumors.** (A) On the left, a representative MCR:eGFP zebrafish with primary tumors along its dorsal side was treated with DMSO control for 2 weeks via oral gavage. On the right, a representative MCR:eGFP zebrafish with primary tumors along its dorsal side was treated with 100 mg/kg Vemurafenib for 2 weeks via oral gavage. The fixed dotted red line represents the tumor area pre-treatment. (B) Average percent change from baseline of tumor area based on pigmentation in a cohort with *n*=6 in the both the DMSO- or Vemurafenib-treated arm. Two-tailed paired *t*-test was performed for statistical analysis. Data represented as mean±s.d. of three replicates. (C) Waterfall plots depict the range of response for the experimental cohorts, DMSO and Vemurafenib. The response was quantified by percent change of tumor area from baseline.

To establish drug efficacy in a non-transplantation model, we directly gavaged transgenic zebrafish harboring primary melanoma tumors. We grouped *Tg(mitfa:BRAF^V600E^); p53^−/−^; mitfa^−/−^*+miniCoopR:eGFP zebrafish and roughly matched based on zebrafish age and tumor size, pigmentation and location. Here, we show a two representative transgenic zebrafish harboring a non-pigmented tumor on the dorsal side, drug-treated daily via oral gavage for two weeks with either DMSO-control or Vemurafenib (100 mg/kg) (Fig. 5A). In comparison with the DMSO treatment, the Vemurafenib-treated primary tumor burden significantly decreased by an average of 70% (*P*<0.001) (Fig. 5B,C). Taken together, this suggests that oral gavage is an effective and inexpensive drug administrative route that allows for user-controlled dose, frequency, and timing of drug delivery.

## DISCUSSION

Our manuscript provides the first proof-of-principle for the use of oral gavage in long-term drug efficacy studies. Although the methodology of oral gavage has been previously described, we introduce two advancements in the technique to allow for multi-day drug studies. Most importantly, we demonstrate oral dosing in the zebrafish model as an effective means to administer a FDA-approved small molecule inhibitor. Although oral gavage has been previously demonstrated as an effective single-intervention technique, challenges of MS-222-related toxicity prevented the gavage application from being used in long-term drug studies. Here, we introduce a novel anesthetic approach combining MS-222 with a second anesthetic, isoflurane, to overcome MS-222-related toxicity. Next, in contrast to a previously published oral gavage apparatus utilizing a 1-cc luerlok syringe, we recommend the use of a 10 μl Hamilton syringe to provide finer control in gavaging small volumes. Previously published studies typically administered 5-10 μl of liquid volume, whereas we reproducibly gavage 2-3 μl of drug or vehicle DMSO without loss of drug volume. Furthermore, we demonstrate that adult zebrafish can tolerate 3 μl of DMSO daily for 2 weeks with minimal toxicity or adverse side effects. Advancements in the anesthetic solution and the physical gavage apparatus significantly improved survival by minimizing MS-222- or DMSO-related toxicity. Taken together, these findings demonstrate the potential of using the zebrafish as a model for drug toxicology and efficacy studies.

In our proof-of-principle efficacy study, we demonstrated successful administration of an orally available FDA-approved BRAF^V600E^ inhibitor, Vemurafenib, to adult zebrafish harboring BRAF-mutant melanoma over the 2-week treatment course. The transplantation model using either zebrafish melanoma cell lines or zebrafish primary melanomas as the donor material resulted in an average 65% tumor reduction (*P*<0.001). Finally, oral gavage of primary melanomas in adult transgenic zebrafish yielded an average 70% tumor reduction (*P*<0.001). The zebrafish model is not only inexpensive to maintain and easily scalable for large treatment cohorts, but recent developments of oral gavage techniques also offer a non-invasive, long-term drug administration platform. Taken together, these advantages allow for future pre-clinical trials in the adult zebrafish.

There are two major causes of injury and trauma during the gavage protocol, and these challenges are compounded when conducting a long-term dosing experiment that requires repeated intervention. The first obstacle is toxicity from long-term exposure to MS-222 as zebrafish do not tolerate daily anesthesia well. Diluting the MS-222 dose and supplementing with a second anesthetic, isoflurane, greatly reduced MS-222-induced toxicity. Zebrafish anesthetized with the MS-222/isoflurane combination have been observed to recover faster compared with zebrafish exposed to a MS-222-only anesthetic.

The second obstacle is contact-induced injury as the soft-tip catheter tubing of the gavage apparatus is inserted into the zebrafish mouth cavity. Minimizing contact of the soft-tip catheter tubing to the mouth will significantly minimize the risk of injury. The use of larger adult zebrafish, at least 3 cm in length, will minimize injury and mortality as the zebrafish can more comfortably accommodate the 22 G soft-tip catheter tubing. Alternatively, larger gauge soft-tip catheter tubing can be used to accommodate for smaller zebrafish. In addition to proper sizing of the gavage apparatus, proper angling and positioning will minimize contact while inserting the soft-tip catheter into the mouth of the zebrafish. Once the zebrafish is positioned vertically and immobilized by the damp sponge, the gavage apparatus should be inserted vertically with maximally a 5° angle towards the researcher. If the user feels any resistance or visualizes motion of the gills, the user should retract the gavage apparatus and reposition.

The development of a drug treatment regimen for Vemurafenib in the adult zebrafish was empirically determined and modeled based on maximizing the duration of drug administration while minimizing any drug-related toxicity or user-induced trauma. Using the current Vemurafenib treatment protocol as outlined, we have not yet observed complete remission with 100% tumor eradication. Instead, we observe that a majority of the zebrafish transplanted with melanoma cell lines or primary tumors are left with a small residual pigmented mass at the injection site when treated for 2 weeks with 100 mg/kg Vemurafenib. The residual pigmented mass cannot be expanded in culture nor can it be transplanted into a secondary recipient. In a study extension, a cohort of Vemurafenib-treated fish harboring a residual pigmented mass was monitored following the two-week treatment time course. We observed growth in the pigmented mass at two months following the end of Vemurafenib treatment, at day 80. It is possible the residual pigmented mass at the end of the treatment time course represents a population of senescent cells unable to engraft in a secondary recipient or proliferate *ex vivo* in culture.

The range of tumor responses to Vemurafenib administered daily for 2 weeks via oral gavage was assessed in adherence to the RECIST algorithm, version 1.1 (Eisenhauer et al., 2009). The RECIST algorithm categorizes drug responses as measured by percent change of the tumor area from baseline over time. Taking advantage of the distinct pigmentation of the melanoma tumor in contrast to normal tissue, digital calipers were used to quantify the tumor area in the zebrafish. As a caveat of the system, the actual tumor response might not be completely captured by a one-dimensional measurement on the most superficial level of the zebrafish intraperitoneal cavity. Additional analysis using small animal PET scanners, for example, might provide a more complete picture of tumor activity *in vivo*.

Although we were unable to observe acquired resistance *in vivo* using the current Vemurafenib treatment protocol as outlined, we provide a strong foundation to build future drug optimization studies. For example, it is logical to rationalize that extending the treatment time course beyond 2 weeks will help achieve the development of acquired resistance *in vivo*. It is also plausible that the dose of Vemurafenib needs to be decreased to allow for a slower selection of drug-resistant clones. Optimization of drug concentration and treatment doses are potential future experiments that can aid in modeling and monitoring acquired resistance *in vivo*. Most importantly, oral gavage provides the technical support required for once-challenging avenues of translational research in the adult zebrafish.

In addition to long-term drug studies, the future of oral gavage will include combination drug treatments *in vivo*. Using therapies for metastatic melanoma as an example, the FDA approved Vemurafenib as an effective single-agent targeted therapy for unresectable metastatic melanoma in 2011. Then in 2014, the FDA approved a BRAF and MEK inhibitor combination therapy as the standard of care. Currently, a majority of human clinical trials in the melanoma field include some form of combination therapy. This indicates the significant need for an *in vivo* cancer model to screen and validate innumerable combination treatments in a timely and financially realistic manner.

Oral gavage in the adult zebrafish is well-suited to be a pre-clinical model to identify and validate anti-cancer combination therapies. Future studies in zebrafish pharmacodynamics and pharmacokinetics for commercially available drugs will undoubtedly aid in the dose optimization for non-toxic drug combinations.

Pre-clinical drug validation using an *in vivo* system is a standard milestone in translational research, and in many cases mouse models are often used. Although murine transgenics and xenograft models are thought to be the gold standards for *in vivo* drug validation, these experiments can be expensive, non-scalable, and poorly reflect the potential of the drugs identified using the zebrafish (Yang et al., 2010). The advent of the oral gavage technique advances the zebrafish as a disease model that has the potential to replace or complement murine studies. Pre-clinical trials in the zebrafish are highly scalable because of their high fecundity and inexpensive zebrafish husbandry. A single researcher can comfortably gavage 2-3 adult zebrafish per minute. Therefore, it is technically and financially feasible to develop clinical trials supporting several cohorts with 100 adult zebrafish dedicated to each arm of the trial. Oral gavage has the potential to be scaled up, as each fish can be gavaged and recovered in less than 30 s post-immobilization. This scalability allows for increasing the number of adult zebrafish in a clinical trial of a given drug, but also allows for small chemical screens to be conducted in the adult zebrafish.

Overall, oral gavage overcomes a long-standing challenge of drug delivery in the adult zebrafish by providing a controlled and non-invasive administrative approach. This has undoubtedly opened up many avenues of drug discovery or validation in the adult zebrafish without outsourcing to mouse models or human cell lines. Most importantly for the future of translational research in the zebrafish model, the efficacy of oral gavage supports the adult zebrafish in small-scale unbiased chemical screens as well as large-scale targeted clinical trials.

## MATERIALS AND METHODS

### Zebrafish mutant and transgenic lines

Zebrafish strains were maintained in accordance to Boston Children's Hospital Animal Research Guidelines. All transplantation experiments used 4-8 months old MHC-matched caspers of at least 1 g in weight and 3 cm in length as measured from mouth to fin. MiniCoopR is a Tol2-based expression vector that contains the zebrafish *mitfa* minigene (promoter, open reading frame, 3′UTR) into the *Bgl*II site of pDestTol2pA2 and in cis a candidate human gene driven by the *mitfa* promoter. Candidate clones were created by Gateway multisite recombination using full-length open reading frames, and include controls eGFP and MEK1DD. MiniCoopR-candidate clone (25 pg) and Tol2 transposase mRNA (25 pg) were microinjected into single-cell *Tg(mitf:BRAF^V600E^); p53(lf), mitf(lf)* embryos. Rescued melanocytes were visible 36-48 hours post-fertilization and these zebrafish have mosaic expression of the candidate gene. Rescued zebrafish were scored for visible tumors weekly beginning at 8 weeks post-fertilization.

### Generation and maintenance of zebrafish melanoma cell lines

ZMEL1 is a zebrafish cell line cultured from a primary zebrafish melanoma tumor overexpressing eGFP in a BRAF^V600E^ and *p53^−/−^* background. The cells were maintained in DMEM (Life Technologies) supplemented with 10% FBS (Life Technologies), 1% Glutamax (Life Technologies), 1% penicillin-streptomycin (Life Technologies) and kept in a sterile 28°C incubator. ZMEL1 cells were washed with sterile PBS (Life Technologies) and trypsinized when harvested for transplantation. Cell numbers were obtained using a hemocytometer and resuspended in sterile PBS and kept on ice awaiting transplant. The ‘PLX^R^’ of ZMEL1-PLX^R^ refers to the isolation of Vemurafenib- (or PLX4032)-resistant clones in the ZMEL1 cell line. This ZMEL1-PLX^R^ resistant line was generated though long-term exposure to 50 nM of Vemurafenib (Selleckchem) for 4 months. Bi-weekly media changes ensured a continuous exposure to Vemurafenib and the generation of drug-resistant clones.

### Excision of zebrafish melanoma tumors

Adult zebrafish harboring primary melanoma tumors were monitored and primary tumors were excised once they had reached 5 mm in diameter. The zebrafish were euthanized according to the Boston Children's Hosptial IACUC protocol. Tumor dissection media was prepared from DMEM/F12 (Life Technologies), 10× penicillin-streptomycin (Life Technologies), 0.075 mg/ml of Liberase (Roche), and the wash media was prepared from DMEM/F12 (Life Technologies), 10× penicillin-streptomycin (Life Technologies), and 15% heat-inactivated FBS (Life Technologies). The tumor was excised with a clean scalpel and razor blade, placed in 2 ml of dissection media, manually disaggregated with a clean razor blade and incubated at room temperature for 30 min. Following manual dissection, 5 ml of wash media was added to the tumor slurry and manually disaggregated one last time. Next, the resuspended tumor cells were passed through a 40 µm filter (BD) into a clean 50 ml tube. An additional 5 ml of wash media was added to the initial tumor slurry and passed through the filter. A final 5 ml of wash media was added to the initial tumor slurry to collect all tumor cells and filtered. Cell numbers were calculated with a hemocytometer and the tubes of resuspended cells were centrifuged at 500 ***g*** for 5 min. The pellet of tumor cells were resuspended in the appropriate volume of PBS and kept on ice prior to transplantation (Heilmann et al., 2015).

### Anesthesia of adult zebrafish

The zebrafish were anesthetized using a duel anesthetic protocol to minimize over-exposure to tricaine in long-term studies. MS-222-only consisted of 4 ml of MS-222 (Western Chemical Incorporated) from a 4 g/l stock in a light-protected bottle into 100 ml of fish water. MS-222/isoflurane #1 refered to 1 ml of MS-222 from a 4 g/l stock and 100 µl of diluted isoflurane into 100 ml of fish water. MS-222/isoflurane #2 refered to 2 ml of the MS-222 from a 4 g/l stock and 100 µl of diluted isoflurane into 100 ml of fish water. The diluted isoflurane was stored at 4°C in a light-protected bottle and composed of undiluted forane (Baxter, NDC-10019-360-40) and ethanol (PHARMCO-AAPER, 111000200) in a 1:9 ratio.

### Intraperitoneal injection

The zebrafish were anesthetized using the 2-step anesthetic protocol detailed above. Once anesthetized, intraperitoneal injections were performed using a beveled, 26S-guaged Hamilton syringe (Hamilton). Anesthetized fish were placed dorsal side up on a damp sponge and stabilized with one hand. Using the other hand, the needle was positioned midline and posterior to the pelvic fin and 500,000 ZMEL1 cells resuspended in PBS injected into the abdominal cavity. Following transplantation, the fish were placed into a recovery tank of fresh fish water and kept off-flow with daily water changes for 7 days to prevent infection. Large and pigmented tumors engrafted and proliferated by 10 days post-transplantation. The syringe was washed in 70% ethanol and rinsed with PBS between uses.

### Oral gavage

Preliminary gavage studies utilized 0.1% fluorescein isothiocyanate (FITC)-dextran (Sigma-Aldrich) and Phenol Red (Sigma-Aldrich) to visualize solution. The zebrafish were anesthetized using the 2-step anesthetic protocol detailed above. Once anesthetized, the zebrafish was propped vertically in a damp sponge and the gavage apparatus dispensed 3 μl of DMSO or Vemurafenib (100 mg/kg). Vemurafenib (Selleckchem), a FDA-approved BRAF^V600E^-specific inhibitor used to treat BRAF-mutant melanoma, was resuspended in DMSO (Sigma) and stored at −80°C for up to 6 months. The zebrafish were then placed into a recovery tank of fresh sterile fish water. The drug regimen was repeated daily for 14 days. The gavage apparatus consisted of a 10 μl Hamilton luer-tip syringe (Hamilton), 22-G Needle (BD), and 22 G soft-tip catheter tubing (Braintree Scientific). The gavage apparatus was adapted from Collymore et al. (2013), who developed a single administration of oral gavage protocol. Immediately following oral gavage, the zebrafish was placed in an isolation tank for recovery and remained isolated for the duration of the treatment period to ensure proper tracking of each subject pre- and post-treatment.

### Imaging and tumor measurements

Photographs of all experimental subjects were obtained at day 10 and day 24 of the experimental timeline. Zebrafish were anesthetized, placed in a dish of fish water, and photographed using a mounted camera (Nikon D3100 with a Nikon AF-S Micro Lens). Tumor area was measured at day 10 and day 24 using a traceable digital caliper (Fisher, 14-648-17). The pigmented tumor area was calculated by the longest measured length and width of the tumor. The drug response was quantified via the change from baseline tumor area using the RECIST (Response Efficacy Criteria in Solid Tumors) guidelines (Eisenhauer et al., 2009). The response rate for experimental cohorts was depicted via waterfall plots, and *t*-test statistics were applied for significance (Gillespie, 2012). Fluorescence imaging of FITC-dextran utilized a Nikon scope with a 0.5× objective lens (Nikon SMZ18-DSRi2)

### Western blot

ZMEL1 tumors from both DMSO- and Vemurafenib-treated cohorts were isolated from euthanized zebrafish at day 20. The bulk tumor was homogenized in 300 μl of RIPA buffer (Sigma-Aldrich) containing cOmplete protease inhibitor tablets (Roche) and phosphatase inhibitors (Sigma-Aldrich) on ice for 20 s. Following homogenization, the sample was kept on a shaker 4°C for 30 min and then spun down at 800 ***g*** at 4°C for 10 min. The supernatant was collected and protein was quantified via DC protein assay (Bio-Rad). The supernatant was combined with 2× laemmli sample buffer with β-mercaptoethanol (BioRad), boiled at 95°C, spun down at 14,000 for 1 min, and 20 μg of protein was loaded onto a 4-15% pre-cast SDS gel (Bio-Rad). The gel was transferred onto a nitrocellulose membrane via iBlot (Invitrogen) and the membrane was blocked in 5% w/v milk in TBS-T for 1 h at room temperature. MAPK activity was assessed via phospho-p44/42 MAPK (ERK1/2) (Thr202/Tyr204) anti-rabbit primary antibody (1:1000, Cell Signaling Technologies #9101), total p44/42 MAPK (ERK1/2) anti-rabbit primary antibody (1:1000, Cell Signaling Technologies #9102), and anti-rabbit IgG HRP-linked secondary antibody (1:2000, Cell Signaling Technologies #7074).

### Zebrafish histology

At day 24, adult zebrafish were euthanized according to the Boston Children's Hospital IACUC protocol. Once euthanized, the zebrafish were placed in a fixative solution of 4% paraformaldehyde and stored at 4°C pending submission to the Dana Farber Histology Core (Boston). The histology core processed the samples and generated three levels of sagittal section through the midline of the pigmented tumor mass. The Dana Farber Histology Core performed H&E staining according to standard protocol.
